# Combining independent *de novo* assemblies to optimize leaf transcriptome of Persian walnut

**DOI:** 10.1371/journal.pone.0232005

**Published:** 2020-04-28

**Authors:** Mohammad Sadat-Hosseini, Mohammad Reza Bakhtiarizadeh, Naser Boroomand, Masoud Tohidfar, Kourosh Vahdati

**Affiliations:** 1 Department of Horticulture, College of Aburaihan, University of Tehran, Tehran, Iran; 2 Department of Horticulture, Faculty of Agriculture, University of Jiroft, Jiroft, Iran; 3 Department of Animal and Poultry Science, College of Aburaihan, University of Tehran, Tehran, Iran; 4 Department of Soil Science, Faculty of Agriculture, Shahid Bahonar University of Kerman, Kerman, Iran; 5 Department of Plant Biotechnology, Faculty of Life Science and Biotechnology, Shahid Beheshti University, Tehran, Iran; National Institute of Plant Genome Research (NIPGR), INDIA

## Abstract

Transcriptome resources can facilitate to increase yield and quality of walnuts. Finding the best transcriptome assembly has not been the subject of walnuts research as yet. This research generated 240,179,782 reads from 11 walnut leaves according to cDNA libraries. The reads provided a complete *de novo* transcriptome assembly. Fifteen different transcriptome assemblies were constructed from five different well-known assemblers used in scientific literature with different k-mer lengths (Bridger, BinPacker, SOAPdenovo-Trans, Trinity and SPAdes) as well as two merging approaches (EvidentialGene and Transfuse). Based on the four quality metrics of assembly, the results indicated an efficiency in the process of merging the assemblies after being generated by *de novo* assemblers. Finally, EvidentialGene was recognized as the best assembler for the *de novo* assembly of the leaf transcriptome in walnut. Among a total number of 183,191 transcripts which were generated by EvidentialGene, there were 109,413 transcripts capable of protein potential (59.72%) and 104,926 were recognized as ORFs (57.27%). In addition, 79,185 transcripts were predicted to exist with at least one hit to the Pfam database. A number of 3,931 transcription factors were identified by BLAST searching against PlnTFDB. Furthermore, 6,591 of the predicted peptide sequences contained signaling peptides, while 92,704 contained transmembrane domains. Comparison of the assembled transcripts with transcripts of the walnut and published genome assembly for the ‘Chandler’ cultivar using the BLAST algorithm led to identify a total number of 27,304 and 19,178 homologue transcripts, respectively. *De novo* transcriptomes in walnut leaves can be developed for the future studies in functional genomics and genetic studies of walnuts.

## Introduction

Persian walnut (*Juglans regia* L.) is an important, profitable species of the genus Juglans [1, 2]. The world’s walnut production and harvest area are 3,747,549 tons and 1,186,399 ha, respectively. China, the United States and Iran are among the largest producers of walnut in the world [3]. Walnut cultivation has a long, archaic history, but opting for breeding programs have only started in the twentieth century. Designing breeding programs using molecular tools have mostly been done in China, the USA, France, Turkey and Iran [4]. The main objectives of breeding programs for the Persian walnut is to achieve specific traits such as late leafing, lateral bearing, good kernel color, high yield, ease of shell cracking, late flowering, early harvest dates and resistance to major diseases. In particular, the prevalence of walnut blight and anthracnose among cultivars has provided good potential for research, which may or may not be coupled with the induction of tolerance to abiotic stresses (e.g. drought and salinity) for rootstocks [2, 4].

*Juglans regia* has a diploid genetic structure, with 2n = 32 chromosomes. It has approximately 606 Mb per 1C genome [5]. Nonetheless, the current information on the genetic modalities of walnut is not as comprehensive as it should be, and the provision of more genetic information can assist researchers in developing efficient breeding strategies for further improvements.

RNA sequencing (RNA-Seq) is a promising application of next generation sequencing (NGS) that has been successfully used for analyzing the entire transcriptome, even for non-model plants such as walnut that lack a complete reference genome [6]. This method enables researchers to identify transcriptome structures, novel transcripts, genes that are differentially expressed, alternative splicing and genetic variants (such as single nucleotide polymorphism) [7]. In addition, the RNA-Seq method can be applied in molecular breeding, especially in relation to research that promote the development of molecular markers [8].

In certain occasions, reference genomes are not available to researchers. A reference transcriptome can be built from RNA-Seq data through *de novo* transcriptome assembly. However, there is a great challenge in terms of accuracy in assembling an RNA-Seq dataset for reliable downstream genetic analysis, which is partly due to the lack of a unique approach to the discipline [9]. Accordingly, there is a clear absence of knowledge about the most suitable assembling algorithms and the best k-mer length for assembling vast amounts of RNA-Seq reads. In this context, there have been few studies about the walnut transcriptome. For instance, more than 49.9 million sequencing reads have been made possible in four organs of walnut (i.e. leaf, bud, female flowers and male flowers). These sequencing reads have been carried out by the Illumina sequencing technology [10], through the process of which one assembly of software is used (Trinity, version 2.0.6). In a previous research, 117,229 transcripts were discovered (N50 of 1955 bp) and, in another study, the Trinity assembly was used for assembling 13,2041,772 reads into 111944 transcripts with a mean length of 1,180 bp and an N50 value of 1,833 [11]. Recently, the biodiversity and plant–microbe interactions in the walnut ecosystem have been a subject of studies using RNA-Seq in California. These were carried out in the context of *de novo* transcriptome studies on walnut [12, 13]. Trinity is the only assembler that has been used for this purpose so far. To the best of our knowledge, no other assembler has been employed to construct an appropriate transcriptome profile for walnut.

This study was designed in an effort to create new RNA-Seq data from Persian walnut. The ultimate goal was to construct a comprehensive annotated transcriptome assembly for the Persian walnut. *De novo* assemblies were constructed with seven independently developed *de novo* transcriptome assemblers having various k-mer sizes. The assemblers were, namely, Bridger (version r2014-12-01) [14], BinPacker (version 1.0.0) [15], SOAPdenovo-Trans (version 1.03) [16], Trinity (version 2.0.6) [17] and rnaSPAdes (version 3.11.1) [18]. Here, there were two widely-used merging approaches which included EvidentialGene (version 2013.07.27; http://arthropods.eugenes.org/EvidentialGene/) and the Transfuse approach (version 0.5.0; https://github.com/cboursnell/transfuse). Considering the applications of these altogether, this research aimed at a combination of all independent assemblies and led to the production of a final assembly. Based on different assembly metrics, 15 transcriptome assemblies were evaluated comparatively so as to find the best *de novo* transcriptome assembly.

## Material and methods

### Plant material

Self rooted clonally propagated walnuts cv. ‘Chandler’ were used for transcriptome sequencing. Eleven two-year old plants were transplanted into 2 liter pots containing peat and perlite (1/1, v/v). Before starting the experiment, the plants were grown in a research greenhouse under controlled environmental conditions (with 16 h light/8 h dark photoperiod for 30 days).

### RNA extraction and cDNA library preparation

The total RNA was isolated from approximately 100 mg of frozen leaf tissue using the RNeasy plant mini kit (Qiagen) according to the manufacturer’s instructions. The concentration of RNA was measured with NanoDrop (Thermo Scientific™ NanoDrop 2000) and the purity of samples were checked on 1% agarose gels for evaluating the 28S and 18S ribosomal RNA bands (28S/18S ratio). If samples had a ratio (28s/18s) of over 1.8 and an OD 260/280 ratio greater than 1.9, they were sent to BGI Co. in China for sequencing. In BGI Co., the RNA integrity number (RIN) was determined using the RIN algorithm of the Agilent Bio analyzer 2100 system (Agilent RNA 6000 Nano Kit, Agilent, Cat No.5067-1511). Only RNA samples that showed a RIN higher than 7 passed the quality test and were used for cDNA library construction. All cDNA libraries were sequenced using a paired-end strategy (read length, 150 bp) on an Illumina HiSeq 2000 platform.

### Read pre-processing

Raw reads were evaluated qualitatively by the FastQC tool (v0.11.5) (http://www.bioinformatics.bbsrc.ac.uk/projects/fastqc/). Low quality bases (Q < 20) and adapter sequences were trimmed using the Trimmomatic (v.0.36) [19] software. Then, clean reads were screened for errors and corrected accordingly using the Rcorrector software (version 1.0.1). The process is actually a Kmer-based error correction of RNA-Seq reads.

### *De novo* transcriptome assembly

All clean and error-corrected reads were assembled into unique putative transcripts by five different assemblers, namely, Bridger (version r2014-12-01) [14], BinPacker (version 1.0.0) [15], SOAPdenovo-Trans (version 1.03) [16], Trinity (version 2.0.6) [17] and rnaSPAdes (version 3.11.1) [18]. It is well known that transcriptome assembly algorithms are strongly affected by k-mer length (i.e. the size of overlapping sequences used in aligning the reads) [20]. Therefore, two k-mer sizes of 25 and 32 were applied for Bridger, BinPacker and Trinity assemblers. The SOAPdenovo-Trans was run with k-mer values of k = 25, 31, 41, 51, 61 and 71. Also, the rnaSPAdes was run with an automatic route of determining the k-mer lengths. Furthermore, CAP3 was used for producing longer consensus transcripts and for reducing the redundancy of contigs obtained through all assemblers. The mentioned production and reduction were performed by setting the minimal identity (%) of the overlap as 95% [21]. For all methods, the minimum transcript length was set at 200 bp. In addition, all transcriptome assemblies that had been assembled using single k-mer assemblers were then merged into a single assembly using two different pipelines, namely, EvidentialGene version 2013.07.27; (http://arthropods.eugenes.org/EvidentialGene/) and the Transfuse v0.5.0 (https://github.com/cboursnell/transfuse). EvidentialGene pipeline (tr2aacds) is usually known to select an ‘optimal’ set of *de novo* assembled transcripts based on the coding potential. The selection is specifically made from a pool of transcript sequences. This pipeline enables researchers to reduce the complexity of the *de novo* transcriptome assemblies by discarding highly similar transcripts, by sequencing fragments and transcripts with low coding potential [22, 23]. On the other hand, the Transfuse can intelligently reconstruct and merge multiple *de novo* transcriptome assemblies [24]. In total, 15 different transcriptome assemblies were compared with each other in the current study.

### Assembly quality assessment

All assemblies (i.e. 13 individual (single k-mer) and two concatenated transcriptome assemblies) were evaluated in terms of accuracy and completeness using typical methods: 1) N50 length (as the shortest contig length representing 50% of the total assembled length) in the transcriptome assemblies, 2) the total number of contigs, 3) the total number of bps in the assembly, 4) the percentage of reads that were mapped back to transcriptomes (or reads being mapped back to the transcriptome RMBT) and 5) the BUSCO analysis (Benchmarking Universal Single-Copy Orthologs, version 2.0.1) for assessing the degree of annotation [25]. The assembly statistics (including N50 length and total number of contigs) were calculated using the TrinityStats.pl from the Trinity package. Bowtie2 (version 2.3.0) was applied in a very-sensitive mode for computing the proportion of clean reads that could be mapped to each assembly [26]. The BUSCO (which is a gene-based quality assessment software) was employed to assess the completeness of the assemblies in terms of gene content. This was performed using the OrthoDB v9.1 ‘embryophyta’ base as a reference, which contains 1,440 BUSCO groups. All 15 transcriptome assemblies were evaluated via the mentioned criteria. The assembly with the best characteristics was selected as the most optimum assembly in terms of performance.

### Functional annotation

The best performing assembly was annotated using different databases as well as bioinformatics tools. The assembled transcripts were translated into putative coding sequences by TransDecoder (version v2.0.1, available at http://transdecoder.github.io) using default parameters. Also, the transcripts were processed through a similarity search against numerous databases at the transcript and peptide levels. To do this, BLASTX (version 2.3.0) was used for comparing the transcripts against the NCBI non-redundant (nr) protein database and against the UniProtKB database (E-value cutoff of 1E-5). Moreover, all predicted proteins (as obtained by TransDecoder) were processed through a BLASTP (version 2.3.0) search against the three databases with an E-value cutoff of 1E-5. The results of BLAST were then processed using ‘analyze_blastPlus_topHit_coverage’ – which is a script of the Trinity package – to assess the fraction of nearly full-length assembled transcripts. In addition, the transcripts were searched for conserved functional domains and transcription factors against the Pfam database and against the plant transcription factor database (PlnTFDB) (version 4.0) using both the HMMER tool (version 3.1b2) [27] and the BLASTn, respectively. The signal peptide and a prediction of the transmembrane domain were estimated by the signalP (version 4.1) and by the tmTMHMM server (version 2.0) programs, respectively. Ultimately, these transcripts were searched against all transcripts of walnut (55,846) which are constantly available in the “Nucleotide” database at NCBI and also available in the genome assembly describing the ‘Chandler’ cultivar of walnut [11].

## Results

### Sequencing the transcriptome in walnut leaf

A high-quality reference-transcriptome-assembly was constructed by extracting the total RNA from 11 leaf samples of Persian walnut seedlings. The transcriptome was sequenced by Illumina HiSeq 2000 platform. In total, 240 million paired-end reads (each having a read length of 150-bp) were generated with a GC content that ranged from approximately 44 to 46%. On average, individual samples yielded 21.8 million (±0.3) reads. After quality trimming, which also included the correction of errors, a total of 231 million high-quality reads were made for *de novo* transcriptome assembly, followed by the relevant analysis. In other words, screening out poor quality reads (Q<20) led to the removal of 3.77% of the raw reads, implying a very high quality of the RNA-Seq data. The results pertaining to the RNA sequencing of the 11 samples were recorded in detail and then summarized (Table 1).

**Table 1:** Summary of transcriptome sequencing of *J*. *regia* obtained from Illumina HiSeq-2000 platform. QC (Quality control).

Sample	Raw result	Trimming results	%GC	Deletion (%)
	Total Sequences	Total Sequences		
1	22326005	21434277	45	3.99
2	22371442	21554690	46	3.65
3	22020370	21032721	46	4.48
4	19106437	18278891	46	4.33
5	22344235	21550592	46	3.55
6	21758291	20881689	46	4.02
7	21637929	20755888	46	4.07
8	22179079	21452220	45	3.27
9	21804304	20912738	46	4.08
10	22187321	21514931	46	3.03
11	22444369	21749718	44	3.09
**Total**	240179782	231118355	45.63	3.77

### Quality evaluation of the walnut leaf transcriptome assemblies

**Fig 1:**
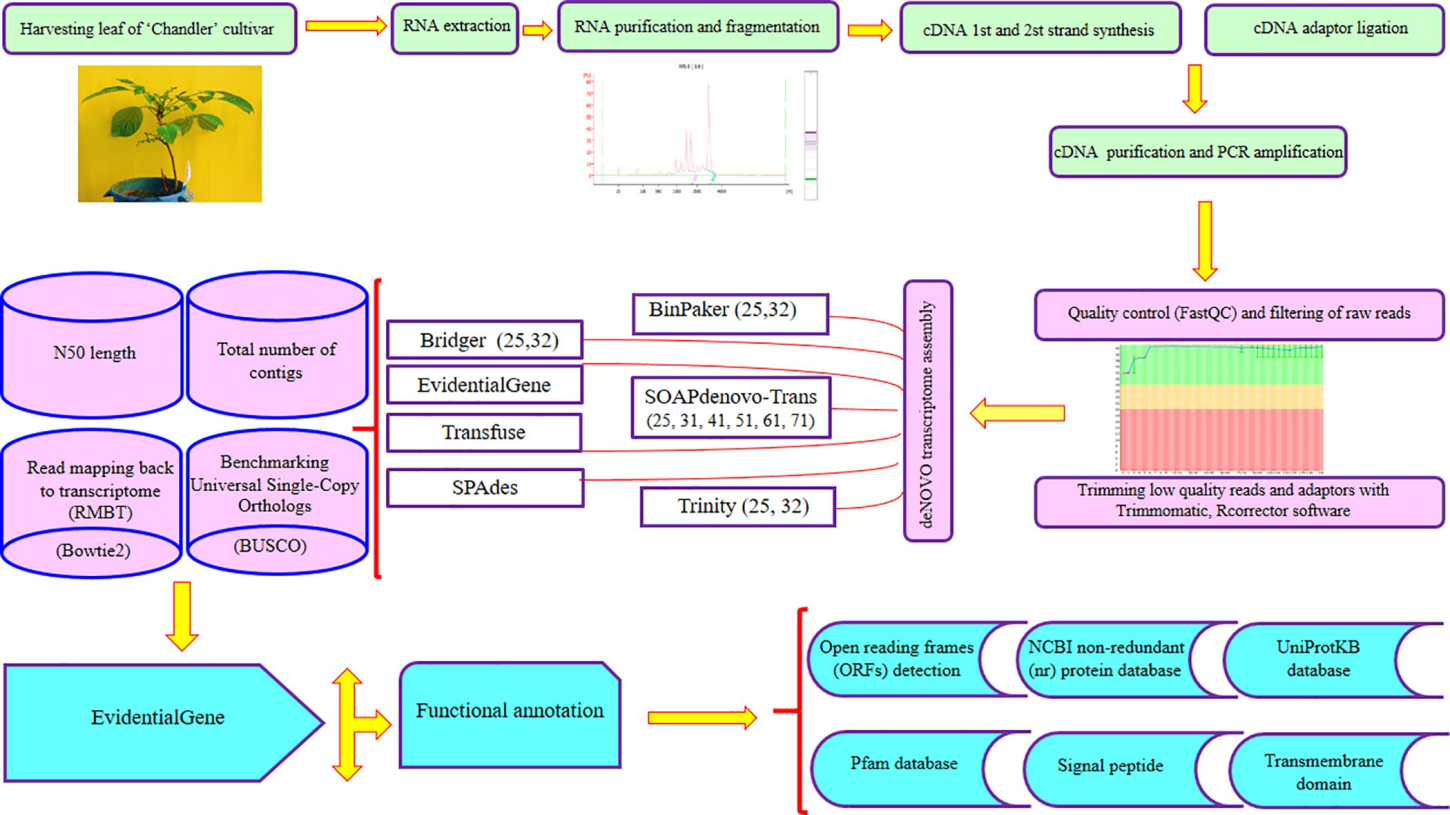
Diagram of the workflow for the walnut leaf transcriptome sequencing, *de novo* assembly and functional annotation. First, mRNA was extracted from leaves of *J*. *regia*, followed by cDNA preparation and construction of the library. Sequencing was done using a paired-end strategy (read length: 150 bp) on an Illumina HiSeq 2000 platform. After quality control and trimming, the *de novo* assembly was constructed via BinPaker, Bridger, SOAPdenovo-Trans, Trinity, SPAdes, EvidentialGene and Transfuse. Then, CAP3 was used for producing longer consensus transcripts and for reducing the redundancy of contigs obtained via all assemblers. The quality of a *de novo* assembled leaf transcriptome was then evaluated by N50 length, the total number of contigs, the number of reads mapping back to the transcriptome (RMBT) and BUSCOs. Finally, the best performing assembly was annotated using different databases, including the UniProtKB database, Pfam database, Signal peptide, ORFs detection, NCBI non-redundant (nr) protein database and the transmembrane domain.

**Fig 2:**
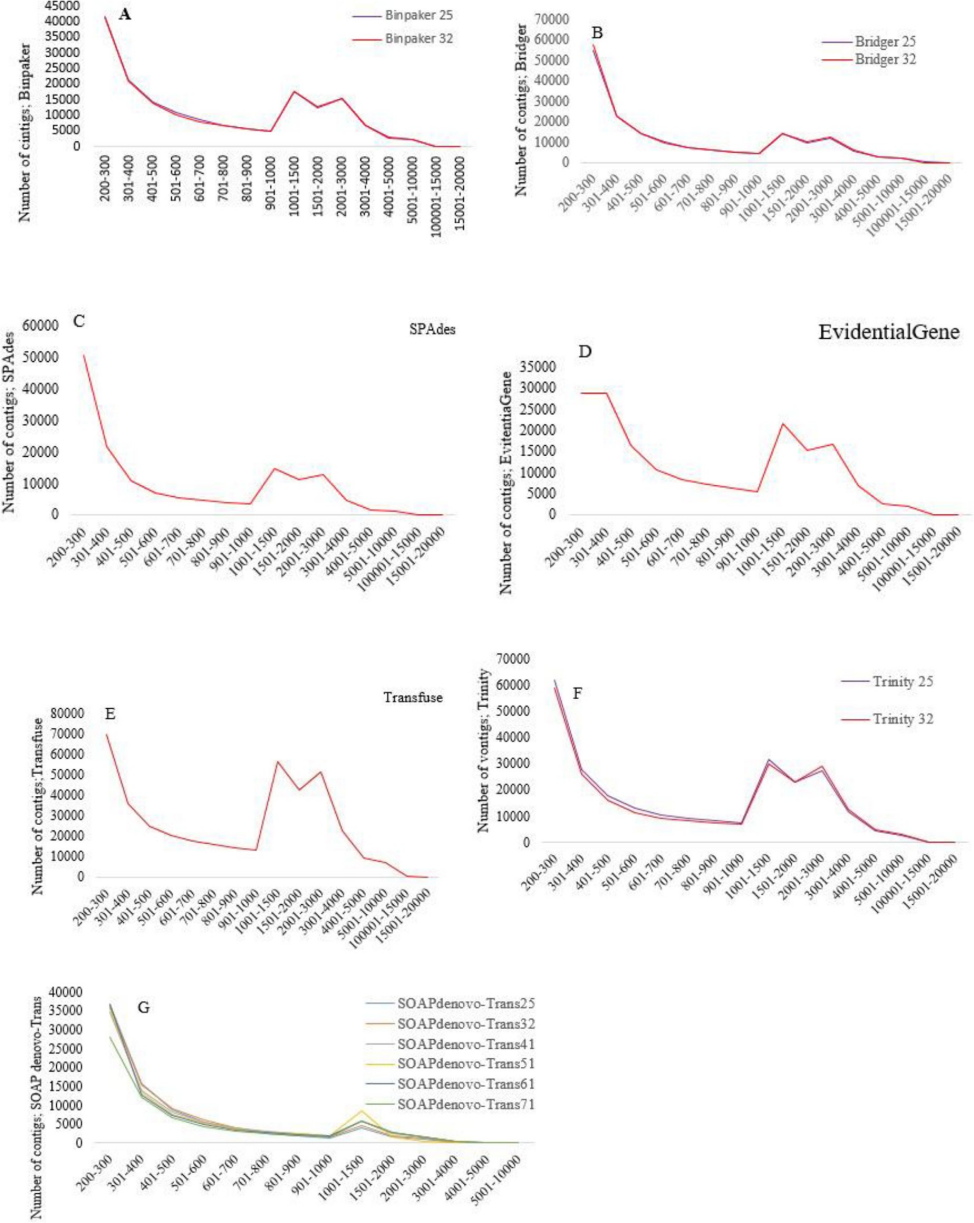
The distribution of contig size and number for all assemblies. A) BinPaker (k-mer; 25 and 32), B) B) Bridger C) SPAdes, D) EvidentialGene, E) Transfuse, F) Trinity (k-mer; 25 and 32), G) SOAP deNOVO-Trans (k-mer; 25, 31, 41, 51, 61 and 71).

**Fig 3:**
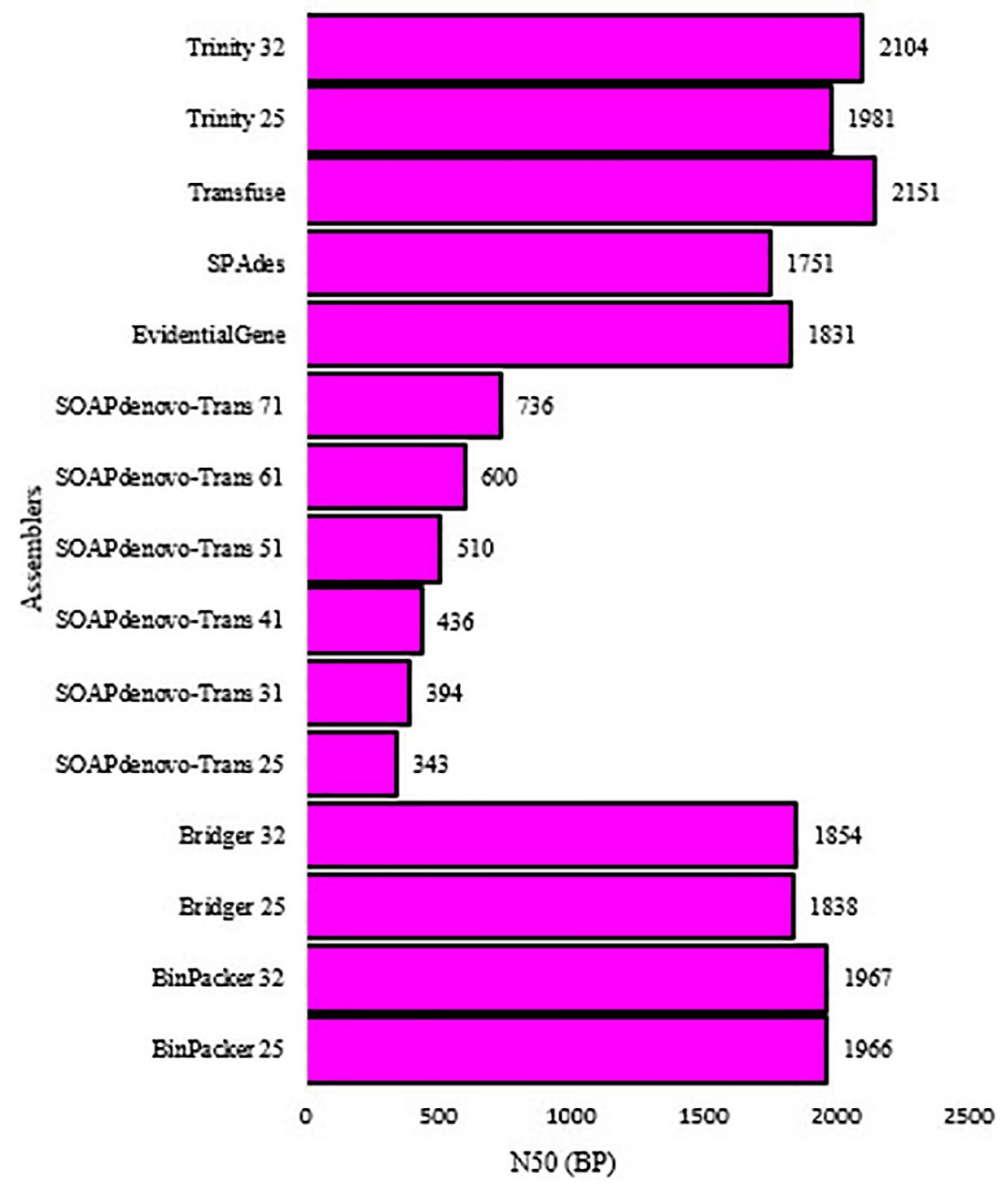
N50 index for each assembler with the k-mer size of walnut leaf transcriptome.

**Fig 4:**
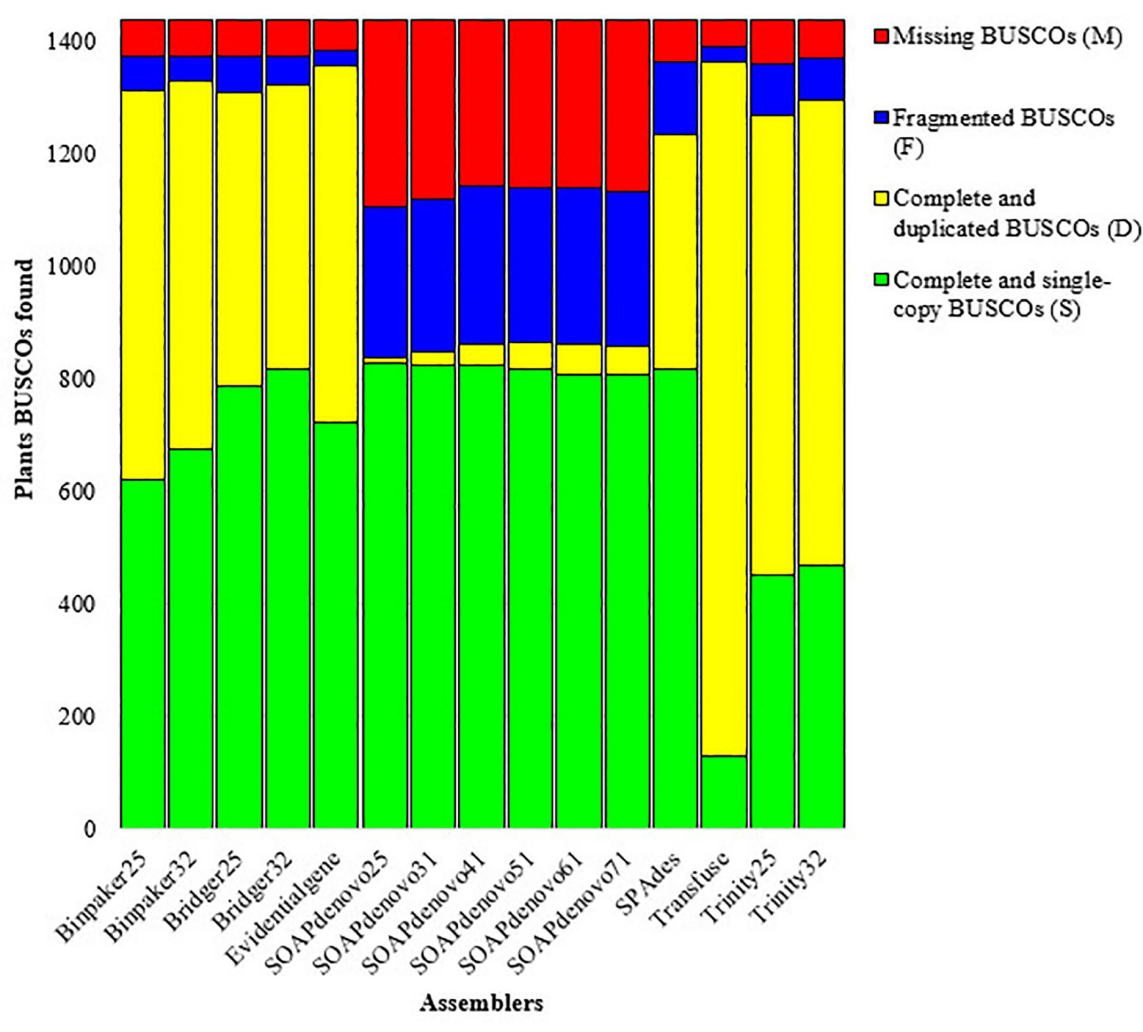
Identifying BUSCOs in each assembler with different k-mer sizes of walnut leaf transcriptome.

Five assemblers (i.e. Bridger, BinPacker, SOAPdenovo-Trans, Trinity and SPAdes) were used along with different k-mer sizes and two merging pipelines (i.e. transfuse and EvidentialGene). Then, 15 *de novo* transcriptome assemblies were created for Persian walnut. Fig 1 shows an overview of the steps concerning the assembly process of pipelines *de novo*. Part of the objective was to find the best transcriptome assembly which differs characteristically from the standard assembly. A summary of the assemblies and their characteristics which are produced by each assembler (including the different k-mer) are shown in Table 2. The number of assembled transcripts varied greatly among the assemblies. Additionally, the number of transcripts decreased when the k-mer lengths of the seven different assemblers increased. In this context, the highest and the lowest number of assembled transcripts were produced by Transfuse (379,406 transcripts) and SOAPdenovo-Trans (k-mer = 71; 88,787 transcripts) methods, respectively. In general, the Transfuse produced about 2 to 5 times more transcripts than the other assemblers. Like the number of transcripts, the output of assemblers varied considerably in terms of the contigs length. Among all assemblies, the Transfuse consistently generated the longest contigs length than any other assemblies, followed by Trinity (k-mer = 32, 1,194.58 bp). The average contig length was 1,282.08 bp. Furthermore, the shortest contig length was measured in SOAPdenovo-Trans, having a k-mer of 25 (247.39 bp). The results of preliminary analysis varied in terms of the contig size in different assemblies (Fig 2). Variations in the contig lengths of the assemblies showed that the contig size frequently occurred within the range of 200-300 bp in all assemblies except when using EvidentialGene (whereby the contig lengths frequently occurred within a range of 300-400 bp). In order to further evaluate the performance of the assemblies, the overall transcriptome sizes were compared. Assemblies from the largest to the shortest transcriptome size were ranked according to the following order: Transfuse (418,444), Trinity (k-mer = 25; 256,396), Trinity (k-mer = 32; 247,229), EvidentialGene (183,191), BinPaker (k-mer = 25; 172,656), BinPaker (k-mer32 =:170,356), Bridger (k-mer = 25; 169,608), Bridger (k-mer = 32; 173,037), SPAdes(154,730), SOAPdenovo-Trans (k-mer = 25; 234,442), SOAPdenovo-Trans (k-mer = 31; 206,075), SOAPdenovo-Trans (k-mer = 41; 179,386), SOAPdenovo-Trans (k-mer = 51; 158,125), SOAPdenovo-Trans (k-mer = 61; 123,329), SOAPdenovo-Trans (k-mer = 71; 88,787). One of the most commonly used metric scales for comparing *de novo* transcriptome assemblies is N50. Assemblies with the longest to the shortest N50 length were, in order of appearance, Transfuse (2,151 bp), Trinity (k-mer 32, 2,104 bp; k-mer = 25, 1981 bp), BinPaker Trinity (k-mer 32, 1,967 bp; k-mer = 25, 1,966 bp), Bridger (k-mer 32, 1854 bp; k-mer = 25, 1,838 bp), EvidentialGene (1,831 bp), SPAdes (1,751 bp) and SOAPdenovo-Trans (k = mer 71 = 736, k = mer 61 = 600, k = mer 51 = 510, k = mer 41 = 463, k = mer 31 = 394 and k-mer 25, 343 bp) (Fig 3). Generally, N50 length of Transfuse was higher than all other assemblies. The implication here is that this method could either remove the small transcripts or merge them together. The results showed that N50 length increased parallel to the increase in the k-mer length in four of the assemblers (i.e. Bridger, BinPacker, SOAPdenovo-Trans and Trinity). Generally, one assembly can be considered as accurate and complete if a large proportion of the reads can map back to the assembly [21]. Apart from the SOAPdenovo-Trans assembler, the other assemblers produced a similar percentage of RMBT, ranging from 99.48% (Transfuse) to 94.25% (BinPaker, k-mer=25). On average, less than 90% of the reads managed to map back to the assemblies generated by SOAPdenovo-Trans. In terms of uniqueness, the highest percentage of mapped reads which uniquely mapped back to the transcriptome were, respectively, the Transfuse (99.48%), Trinity (k-mer=32, (98.8%); k-mer=25, (98.26%)), SPAdes (97.94%), Bridger (k-mer=32, (96.73%)), BinPaker (k-mer=32, (96.72%)), EvidentialGene (96.06%), Bridger (k-mer=25, (94.13%)) and BinPaker (k-mer=25, (94.11%)) (Table 3). The other assemblies (except the SOAPdenovo-Trans) were similar in terms of their percentages of reads which uniquely mapped back to the transcriptome. These appeared to range between specific percentages: Transfuse (99.39 to 99.64%), Trinity (k-mer=32, (98.68 to 98.92%); k-mer=25, (98.09 to 98.36%)), SPAdes (97.72 to 98.04%), Bridger (k-mer=32, (96.46 to 96.93%)), BinPaker (k-mer=32, (96.43 to 96.80%)), EvidentialGene (95.57 to 96.45%), Bridger (k-mer=25, (93.67 to 94.35%)) and BinPaker (k-mer=25, (93.63 to 94.33%)). Similar to earlier observations, the SOAPdenovo-Trans showed the worst performance in this case and had the lowest percentage of reads which were uniquely mapped back, ranging from 73.84% to 91.23%. In addition to RMBT, it is quite necessary to achieve a measure of completeness in terms of the number of genes that are detected. For this purpose, transcriptome assemblies for walnut were accessed based on their completeness, contiguity and accuracy, as measured by the BUSCO approach, whereby the assemblies displayed different scores. While the Transfuse and EvidentialGene methods preformed best in the BUSCO score, SOAPdenovo-Trans operated badly in the category. Out of the 1,440 single-copy ortholog genes that are common in plants, 94.8% (1,364 BUSCOs) and 94.3% (1,358 BUSCOs) were identified in Transfuse and EvidentialGene assemblies, respectively. Transfuse performed best in terms of having the least number of missing BUSCOs (48, 3.3%), followed by EvidentialGene (54, 3.8%). Then, with regard to fragmented transcripts, Transfuse (1.9%) and EvidentialGene (1.9%) assemblies contained a minimum number of fragmented BUSCOs, while each of the other assemblies (except SOAPdenovo-Trans assemblies) contained BinPaker (4.3%), Bridger (3.95%), SPAdes (8.8%) and Trinity (5.85%) within the fragmented transcripts. SOAPdenovo-Trans assemblies contained the highest number of fragmented (19.08% on average) and missing (21.45% on average) BUSCOs (Fig 4).

**Table 2:** Statistics of the leaf transcriptome pre-assemblies and the final *de novo* assembly in self rooted Persian walnut ‘Chandler’.

Assembler	BinPacker		Bridger		SOAPdenovo-Trans						SPAdes	Trinity		Transfuse	Evidential Gene
k-mer	25	32	25	32	25	31	41	51	61	71		25	32		
NS	185380 854	182751 763	157007 643	160526 566	57999046	58620636	58610033	55903363	50016501	43360309	141061136	289843659	295334827	536477927	193422472
MCL (bp)	584	581	447	441	160	177	191	206	237	294	428	673	723	854	602
ALC (bp)	1073.7	1072.7	925.7	927.7	247.4	284.5	326.8	353.5	405.6	488.4	911.7	1130.4	1194.6	1282.1	1055.9
N50 (bp)	1966	1967	1838	1854	343	394	436	510	600	736	1751	1981	2104	2151	1831
TG	132929	135394	142382	142382	234442	206075	179386	158125	123329	88787	133757	129613	129020	379406	183191
TT	172656	170356	169608	173037	234442	206075	179386	158125	123329	88787	154730	256396	247229	418444	183191
BUSC Os (%)	91.3	92.3	90.9	91.9	58.2	58.9	59.9	60.0	60.0	59.8	85.9	88.2	90.0	94.8	94.3

NS (Number of sequences), MCL (Median contig length), ALC (Average length contig) TG (Total gene), TT (Total transcripts).

**Table 3:** Percentage of reads mapped back to the walnut leaf transcriptome (RMBT).

								Assembler							
Sample	BinPacker/25	BinPacker/32	Bridger/25	Bridger/32	Evidentialgene	SOAPdenovo-Trans/25	SOAPdenovo-Trans/31	SOAPdenovo-Trans/41	SOAPdenovo-Trans/51	SOAPdenovo-Trans/61	SOAPdenovo-Trans/71	SPAdes	Transfuse	Trinity/25	Trinity/32
**1**	94.04%	96.72%	94.05%	96.74%	95.57%	75.78%	83.74%	90.60%	92.80%	93.06%	93.62%	97.88%	99.64%	98.36%	98.91%
**2**	94.27%	96.80%	94.29%	96.84%	95.81%	74.58%	82.48%	89.10%	91.39%	92.13%	93.04%	98.02%	99.50%	98.30%	98.92%
**3**	94.26%	96.65%	94.27%	96.68%	96.19%	75.24%	82.43%	88.53%	90.62%	91.25%	91.90%	97.92%	99.39%	98.19%	98.75%
**4**	94.30%	96.72%	94.31%	96.74%	96.36%	75.33%	82.50%	88.55%	90.67%	91.33%	92.02%	97.97%	99.39%	98.16%	98.77%
**5**	94.33%	96.91%	94.35%	96.93%	95.88%	73.02%	81.34%	88.40%	90.76%	91.51%	92.35%	97.98%	99.51%	98.34%	98.92%
**6**	94.32%	96.77%	94.33%	96.79%	96.45%	75.15%	82.49%	88.69%	90.91%	91.56%	92.25%	98.04%	99.47%	98.22%	98.85%
**7**	94.14%	96.71%	94.16%	96.72%	96.34%	74.54%	82.04%	88.49%	90.72%	91.36%	92.05%	97.98%	99.48%	98.35%	98.87%
**8**	93.76%	96.43%	93.80%	96.46%	95.75%	72.20%	80.02%	86.80%	89.09%	89.71%	90.37%	97.98%	99.50%	98.09%	98.84%
**9**	93.98%	96.66%	94.01%	96.67%	96.50%	73.08%	80.15%	86.41%	88.40%	88.68%	89.24%	97.87%	99.41%	98.30%	98.68%
**10**	94.14%	96.70%	94.16%	96.71%	95.67%	74.17%	81.70%	88.41%	90.56%	91.21%	91.92%	98.01%	99.52%	98.12%	98.88%
**11**	93.63%	96.80%	93.67%	96.79%	96.12%	69.18%	76.23%	82.31%	84.25%	83.99%	84.78%	97.72%	99.50%	98.45%	98.73%
**Mean RMBT**	94.11%	96.72%	94.13%	96.73%	96.06%	73.84%	81.37%	87.84%	90.02%	90.53%	91.23%	97.94%	99.48%	98.26%	98.83%

### Functional annotation

After evaluating the different *de novo* assemblies, EvidentialGene was selected as the final assembly and was utilized for the functional annotation analysis. Out of 183,191 transcripts, a total of 111,451 transcripts were annotated using one or more databases. Based on the initial transcript sequences and the TransDecoder tool, 104,926 ORFs were predicted. In total, 84,384 transcripts and 71,314 protein sequences shared similarities when compared against the UniProtKB database using the BLASTx and BLASTp searches, respectively. When aligned against the NCBI nr database, the number of significant homologous transcripts and protein sequences increased to 109,413 and 85,523, respectively. Protein sequences with little similarity at the sequence level can share conserved domains, and thus a domain-based annotation was performed. Accordingly, 79,185 transcripts were found with at least one hit to the Pfam database. The final assembly included 79,185 transcripts with at least one hit to the Pfam database. Moreover, 3,931 transcription factors were identified by BLAST searching against PlnTFDB. In comparing the transcripts with the Rfam database, 882 significant matches were found. On the other hand, 6,591 and 92,704 of the predicted peptide sequences contained signaling peptides and transmembrane domains, respectively. Finally, a total number of 27,304 and 19,178 of transcripts revealed similarities based on BLAST search against all transcripts of the walnut and of the published genome assembly for the ‘Chandler’ cultivar, respectively (Table 4, S1 and S2 Tables).

**Table 4:** Functional annotation summary of walnut leaf transcriptome.

Category	No. of transcripts)
Total transcripts	183,191
Predicted proteins	109,413
Predicted ORFs	104,926
Pfam	79,185
SignalP	6,591
Rfam	882
Transcription factor	3931
ATNDW*	27,304
ATGAPCh**	19,178

*Assembled transcripts searched against all nucleotides of walnut

** Assembled transcripts searched against the published ‘Chandler’ genome assembly

## Discussion

In plant species without a published genome or with an incomplete sequenced genome, such as walnut, the occurrence of *de novo* transcriptome assembly facilitates the study of transcriptomes and that of relevant genes of expression. However, lacking an optimum standard for *de novo* transcriptome assembly can entail several challenges and limitations. The current study was an attempt to alleviate those challenges and reduce the limitations. Specifically, it involved identifying a transcriptome reference in walnut leaves, one that is likely to be complete and in detail when compared to the 15 transcriptome assemblies that were generated with five *de novo* assemblers and two merging approaches. Additionally, the best assembly was selected based on the quality of assembly in terms of N50 length, the total number of contigs, the total number of bps in the assembly, RMBT and BUSCOs. Our analyses took advantage of the 11 RNA-Seq libraries with increasing numbers of Illumina paired-end reads. In total, a comprehensive strategy for walnut transcriptome assembly was applied. This can be applied to settings that require the reconstruction of high quality transcriptome in non-model species other than the walnut.

After trimming the raw reads, 96.23% of the reads (i.e. 231 million) were used for *de novo* transcriptome assembly. Previous studies have reported that the range of GC content in different samples has important effects on transcriptome assemblies and on the variation of the GC content among the libraries which can lead to shorter assembled transcripts [28, 29]. Here, the GC content in all samples ranged approximately from 44 to 46%. This finding is in agreement with a previous study that showed a GC content of 46.6% in *Juglans hopeiensis* [30].

In line with previous studies, the results showed that there is a variation among the metrics of the assemblies. Previous research indicated that different assembly methods generate significantly different assemblies [20, 31]. However, apart from SOAPdenovo-Trans, most of the assemblers that were used in the current research were equally good and, in many aspects, proved to be competitive and comparable to each other. Since SOAPdenovo-Trans is mentioned as an exception, the assemblies being generated thereof were excluded from further evaluation. Generally, the quality metrics were fairly even among the top performing assemblies (i.e. all assemblies except the SOAPdenovo-Trans) in terms of N50 and contig lengths. All of the top performing assemblies showed an N50 length of more than 1,800 bp with similar contig length distributions. Previous research demonstrated that N50 in the walnut transcriptome is 1,833 bp when processed through Trinity [11]. In another study on *Juglans mandshurica*, N50 length was reportedly 1,863 bp [13]. The average contig length was higher than 900 bp in all of the top performing assemblies, which is comparable to previous studies on plants such as *J*. *hopeiensis* (731 bp) [30], *Camelina sativa* L. (1198 bp) [32], *Pinus contorta* (715 bp) [33] and *Lens colinaris* (770 bp) [34]. The current results are in line with earlier studies, even though N50 and contig lengths cannot be used for selecting the best assembly. Basic parameters such as N50 and contig lengths can be used in genome assembly, but it is well known that these parameters are inappropriate for the evaluation of transcriptome assembly, mainly because the expected transcript lengths remain unknown in some species. Furthermore, longer transcripts or larger total assemblies do not necessarily indicate a better transcriptome assembly [35]. In addition, the available scientific literature suggests that N50 values can be artificially changed based on the defined k-mer size or based on the minimum contig length [36]. On the other hand, the average of RMBT in this study appeared to be more than 94% in all assemblies that showed high levels of performance. It is worth noting that the RMBT percentage in this study turned out to be higher than those reported in previous studies, thereby indicating a high quality of the assemblies. For example, the RMBT is reportedly 89.93% in pistachio (*pistacia vera* L) [37] and 83.68% in almond (*Prunus dulcis* Mill.) [38]. Despite the fact that the three mentioned metrics (i.e. N50, contig length and RMBT) can evaluate the assemblies in different aspects, understanding the biological differences between the assemblies becomes quite difficult when evaluations are based on these metrics. Accordingly, BUSCO can be considered as an important measure of performance in studies of this kind. BUSCO is capable of searching against a database of highly conserved single-copy ortholog genes. It checks whether each BUSCO group is either complete, duplicated, fragmented or missing in the transcriptome assembly. Among the assemblies, the merging pipelines performed better as Transfuse (94.8%, with 1364 complete BUSCOs) and as EvidentialGene (94.3%, with 1358 complete BUSCOs), thereby yielding the highest BUSCO scores. These results accord closely with previous studies which reported a better performance of merged transcriptome assemblies generated from multiple assemblies [39]. In addition, the number of duplicates appeared to be high in the Trinity, but had a lower score of 88.2% (with 1,270 complete BUSCOs in kmer = 25) and of 90% (with 1,296 complete BUSCOs in kmer = 32) when compared to EvidentialGene and Transfuse. Taken together, each of the assemblers has different algorithms and can potentially identify a small number of unique transcripts relative to each other [20, 40]. Thus, it might be worthwhile to combine assemblies that are generated by different assemblers, which might reconstruct a more complete transcriptome reference. However, these methods are more complicated to perform than the straightforward *de novo* assemblers like Trinity in terms of time and resources.

Of the merged assemblies, Transfuse showed a slightly better performance based on the final score of BUSCO, but it produced more than double the number of contigs generated by the other merging assembler, indicating that this tool produced more redundancy relative to EvidentialGene. Also, this finding was not in line with a few previous studies on walnut and on other close plants [11, 37, 41]. In this regard, the number of duplicated copies (based on BUSCO analysis) was higher in Transfuse (85.8%) than in EvidentialGene (44.1%), which can be considered as a weakness of Transfuse. In contrast, the percentage of complete and single copy scores of BUSCOs by EvidentialGene was higher than by Transfuse, which can be more handy in downstream analysis. On the other hand, Transfuse (92.49%) had 27.98% more reads than EvidentialGene (64.50%). These reads were aligned concordantly more than once (S3 Table). It reiterates that the assembly generated by the Transfuse method contained more redundancy than when generated by EvidentialGene. This can lead to a bias in downstream gene expression and enrichment analysis. Therefore, a higher percentage of reads being uniquely mapped back and the lower percentage of duplicated BUSCO scores can make EvidentialGene be selected as the final transcriptome, which might be preferable to other downstream analyses. Of the 193,422,472 transcripts produced by EvidentialGene, 61% were annotated using one or more databases, which is better than those reported previously on walnut and on the other close plants. In another report on walnut, only 22.26% of the transcripts were annotated [11]. In the current study, 104,926 ORFs were predicted, while another study on walnut predicted a total of 35,836 ORFs out of a total of 111,944 transcripts [11]. In mango (*Mangifera indica* L.), 80,969 transcripts were reported, out of which 33,142 ORFs were predicted [42]. The current study revealed 3,931 transcription factors when the transcripts were searched by BLAST against PlnTFDB (S4 Table). Some of these transcription factors were identified as MYB, WRKY, bHLH, NAC and bZIP. To the best of our knowledge, only seven studies have so far investigated walnut transcriptome assemblies. Those studies are publicly available and can be compared with our study. The past seven studies had used Trinity alone and by default parameters so as to construct *de novo* assemblies [10, 11, 12, 13, 30, 43, 44]. In addition, none of the studies made use of BUSCOs to evaluate the quality of assemblies. Here, however, we evaluated the walnut transcript according to the relevant database along with BUSCOs. Previous results had shown that 1,440 single-copy ortholog genes are common in plants by 89.9% (indicating 1,294 BUSCOs). This is while the current study led to the identification of 94.8% (with 1364 BUSCOs) and 94.3% (with 1358 BUSCOs) by Transfuse and EvidentialGene.

## Conclusion

The purpose of the current study was to construct a complete transcriptome assembly in walnut leaves. This involved using a range of tools and k-mer values. To the best of our knowledge, this is the first study in which different assemblers were evaluated with a range of k-mers to create a *de novo* transcriptome assembly in walnut. Combining assemblies that are generated by straightforward *de novo* assemblers can lead to the construction of a more comprehensive transcriptome assembly. In addition, based on various quality metrics of assembly, the transcriptome assembly which is produced by EvidentialGene can be prioritized over other assemblies in walnut leaves. In summary, the reference transcriptome assembly which was generated by EvidentialGene included 183,191 transcripts with an N50 length of 1,831 bp, among which 64,702 transcripts were longer than 1 kb.

## Supporting information

S1 Table (XLSX)

S2 Table (XLSX)

S3 Table (XLSX)

S4 Table (XLSX)

S5 Table (XLSX)

S1 Text (TXT)
